# Nonmelanoma skin cancer risk awareness in azathioprine‐treated myasthenia gravis patients

**DOI:** 10.1002/brb3.396

**Published:** 2015-09-15

**Authors:** Iain J. McGurgan, Christopher McGuigan

**Affiliations:** ^1^ Department of NeurologySt. Vincent's University HospitalElm ParkDublin4Ireland

**Keywords:** Awareness, azathioprine, immunosuppression, myasthenia gravis, nonmelanoma skin cancer

## Abstract

**Objectives:**

Increased rates of NMSC (nonmelanoma skin cancer) have recently been reported in people with MG (myasthenia gravis) receiving azathioprine treatment. Guidelines on azathioprine for patients with dermatological and gastrointestinal disorders stress the importance of NMSC risk awareness and prevention. The aim of this study is to assess whether MG patients are being informed of this risk.

**Methods:**

Clinical records of patients with MG attending a university hospital neurology clinic were reviewed. Data on patient demographics, clinical presentation, diagnostic tests, azathioprine treatment, development of NMSC, and counseling regarding NMSC risk were recorded.

**Results:**

Sixty‐nine MG cases were identified, median age 58 years (range 20–90). Forty‐two (60.9%) had received azathioprine at some point with a mean cumulative dose of 235.5 g (range 9.1–972.8 g). Skin cancer risk and prevention advice provision was documented in 3 (7.1%) azathioprine‐treated patients. Five patients developed histologically confirmed NMSC of whom all were treated with azathioprine (incidence rate of 24.9 per 1000, 16 times higher than expected). Documented advice on other safety issues such as regular blood test monitoring was found in 33 (78.8%) azathioprine‐treated cases.

**Conclusions:**

Preventative measures such as daily sunscreen use have been shown to reduce the incidence of NMSC in the general population. The results of this study demonstrate a very low rate of advice provision about NMSC risk in azathioprine‐treated MG patients and the need for increased awareness among treating neurologists and patients.

## Introduction

Definitive treatments of MG (myasthenia gravis) are focused on suppressing the autoantibody‐mediated damage to the postsynaptic neuromuscular junction. Treatment guidelines recommend immunotherapy to induce and maintain remission when symptomatic treatment with acetylcholinesterase inhibitors is insufficient, with azathioprine regarded as first‐line therapy (Skeie et al. 2010). Azathioprine is a derivative of thioguanine (a purine mimic antimetabolite) that is rapidly converted to 6‐mercaptopurine, which inhibits DNA and RNA synthesis and disrupts T‐cell function (Elion 1989). This drug is usually well tolerated but nausea, hypersensitivity, pancreatitis, hepatitis, and myelotoxicity are all well‐recognized side effects (Meggitt et al. 2011).

There is also an established risk of malignancy, particularly lymphoma, associated with long‐term azathioprine treatment. Recently, increased rates of NMSC (nonmelanoma skin cancer) have been reported in organ transplant recipients and IBD (inflammatory bowel disease) patients receiving high‐dose azathioprine, and in 2014 this observation was corroborated for the first time in a population of MG patients (Pedersen et al. 2014). Current guidelines on the treatment of dermatological and gastrointestinal disorders with azathioprine stress the importance of making patients aware of this risk and providing information on preventative measures (Meggitt et al. 2011; Mowat et al. 2011). The aim of this study is to assess the incidence of NMSC in an azathioprine‐treated MG cohort and the frequency of advice provision on NMSC risk and preventative practices at a university hospital neurology department.

## Methods

All patients with confirmed MG attending a university hospital neurology department were identified. Clinical records, prescription copies, and computerized investigation records of patients attending the hospital's MG clinic and all those with a diagnosis code of MG in the hospital's inpatient registry were obtained. Those cases which were not followed up on‐site or had since died, those which on the basis of negative/inconclusive investigations were deemed unlikely to have MG and those with insufficient information available to derive conclusions about diagnosis and management were excluded. In the remaining cases, data on patient demographics, clinical presentation, diagnostic tests, azathioprine treatment, adverse effects encountered on azathioprine, development of NMSC, counseling regarding NMSC risk, and dermatology clinic attendance were recorded. Prednisolone was not considered as immunotherapy for MG for the purpose of this study. The cumulative azathioprine dose (in grams) was calculated for each patient using documentation of treatment initiation and termination, dose changes, and treatment interludes. Total exposure time was calculated from the time of commencement until the index date (accounting for breaks in treatment), and azathioprine prescribed within 1 year of the index date was categorized as current usage at the same dosage unless there was evidence to suggest treatment had been altered or discontinued. Those patients who had received azathioprine for less than 6 continuous months at any stage in their treatment were not included in the calculation of cumulative azathioprine dose, treatment duration, or NMSC incidence rate. A long duration of azathioprine treatment was defined as use for more than 5 years and high exposure was defined as a cumulative dose in excess of 150 g. The incidence rate of NMSC in the azathioprine‐exposed study population was calculated and compared to the NMSC incidence in the general Irish population derived from the 2013 National Cancer Registry Ireland age‐standardized incidence rates 1994–2011 (Cancer trends No 20, 2013).

Approval for this research was granted by the institution's clinical audit committee. This study was exempt from the requirement for ethical approval.

## Results

Sixty‐nine MG cases were identified. The mean duration of illness (the time since confirmed diagnosis) was 11.1 years. Additional characteristics of the subjects are highlighted in Table 1.^1^ Forty‐two (60.9%) patients had received azathioprine at some point since diagnosis, with 34 of these having been maintained on treatment continuously for at least 6 months. The mean cumulative dose of those treated for 6 months or more was 235.5 g (range 9.1–972.8 g) with a mean treatment duration of 6.1 years (range 0.5–20.7 years). Seventeen (50%) had received a high (>150 g) cumulative dose and 16 (47.1%) had long‐term (>5 years) total azathioprine exposure. Twenty‐two (52.3%) of those patients who had ever received azathioprine discontinued treatment, eight of these within 6 months of therapy commencement. Twenty patients discontinued due to adverse effects and the remaining two due to lack of a demonstrable clinical response and disease remission, respectively. Adverse effects associated with azathioprine use were documented to have occurred in 28 (66.7%) of those patients ever treated (Table 2). In those patients who discontinued azathioprine, nine initiated alternative immunotherapy (five with methotrexate and four with mycophenolate mofetil). Seventeen (24.6%) patients underwent a thymectomy at some stage since diagnosis.

**Table 1:** Characteristics of study participants

Sex	Frequency	Percentage
Female	39	56.5
Male	30	43.5

Age	Median	Range
(Years)	58	20–90

Initial presentation	Frequency	Percentage of total
Ocular symptoms	36	52.2
Combination of ocular/bulbar/limb weakness	21	30.4
Bulbar weakness	6	8.7
Limb weakness	4	5.8
Fatigue	1	1.4
Thymic mass	1	1.4

Antibody status	Frequency	Percentage of those tested
AChR antibody	43	66.2
Antibody negative	17	26.2
Anti‐striated muscle antibodies	9	13.8
MuSK antibody	4	6.2
Not tested	4	—

AChR, acetylcholine receptor; MuSK, muscle‐specific receptor tyrosine kinase.

**Table 2:** Adverse effects attributed to azathioprine treatment. Two patients reported multiple side effects

Adverse effect documented	Frequency	Percentage of those ever treated with azathioprine	Frequency of discontinuation of azathioprine due to this adverse effect	Percentage of discontinuation of azathioprine due to this adverse effect
Cytopenias	10 (leukopenia 5, anemia 3, thrombocytopenia 1, pancytopenia 1)	23.8	6	27.3
LFT derangement	7	16.7	5	22.7
NMSC	5	11.9	3	13.6
Gastrointestinal symptoms	4	9.5	3	13.6
Flu‐like symptoms	2	4.8	1	4.5
Hypersensitivity reaction	1	2.4	1	4.5
Seizures	1	2.4	1	4.5

LFT, liver function tests; NMSC, nonmelanoma skin cancer.

Skin cancer risk and prevention advice provision was documented in 3 (7.1%) of azathioprine‐treated patients before or at the time of first prescription. No patients had documented evidence of NMSC prior to diagnosis with MG, however one patient had been attending the dermatology clinic with a pre‐established diagnosis of lichen simplex chronicus (this patient was one of the five to later develop NMSC). Five patients developed histologically confirmed NMSC (three SCC [squamous cell carcinoma], four BCC [basal cell carcinoma], including two patients who had both BCC and SCC) (Table 3). All cases of confirmed NMSC had prior azathioprine exposure, at a mean cumulative dose of 357.2 g (range 127.8–793.9 g) over a mean treatment duration of 10.1 years (range 5.6–15.0 years). No cases of NMSC were identified in the nonazathioprine‐exposed group or in those who received azathioprine for less than 6 months of continuous treatment. The incidence rate of NMSC in the azathioprine‐exposed population was 24.9 (95% confidence interval 10.5–59.3) per 1000 person‐years of exposure. The expected number of cases in this group over the total treatment period (based on the nationwide incidence rate) is 0.31. Documented advice on the importance of regular blood test monitoring was found in 33 (78.8%) azathioprine‐treated cases. TPMT (thiopurine methyltransferase) enzyme activity testing was carried out in 18 (42.9%) patients prior to azathioprine initiation or dose escalation and was within normal limits in all cases. Skin cancer safety advice was documented to have been provided by dermatologists in four of seven azathioprine‐treated MG patients seen by dermatologists for any reason, however in each case this was after the diagnosis of NMSC. Additionally, two patients (both receiving azathioprine treatment) attended the dermatology clinic with actinic keratosis.

**Table 3:** Characteristics and management of the five NMSC cases

Case	NMSC	Management	Azathioprine/follow‐up
Case 1	Moderately differentiated SCC on left leg, 20 mm in maximum diameter, depth 3.6 mm	Excised with a 10‐mm margin	Azathioprine stopped, regular dermatology follow‐up
Case 2	BCC right forehead	Mohs excision	Azathioprine continued, dermatology follow‐up
Case 3	BCC left infraorbital region	Mohs excision	Azathioprine stopped, methotrexate dose increased (previously treated with 25 mg methotrexate weekly for rheumatoid arthritis), regular dermatology follow‐up
	Nodular BCC right medial canthus	Mohs excision	
	Moderately differentiated SCC right eyebrow, right parotid SCC (suspected metastasis)	Excision, right parotidectomy (1 mm margin) and adjuvant radiotherapy	
Case 4	Moderately differentiated SCC scalp, depth 4.9 mm	Excised	Azathioprine dose reduced, annual dermatology review
	BCC right forehead	Mohs excision	
Case 5	Multiple BCC's: scalp, forehead, nose	Mohs excisions	Azathioprine stopped

SCC, squamous cell carcinoma; BCC, basal cell carcinoma; NMSC, nonmelanoma skin cancer.

## Discussion

Despite being envisaged initially as a chemotherapeutic agent and pioneering the treatment of acute lymphoblastic leukemia, azathioprine is now considered a carcinogen and classified as such by the International Agency for Research on Cancer (Karran and Attard 2008). In its antiproliferative effects on active lymphocyte populations resides both its immunomodulatory efficacy and its promotion of neoplasia, the latter via defective T‐cell immunosurveillance (Aithal and Mansfield 2001). Prolonged use of azathioprine in solid‐organ transplant recipients has been demonstrated to increase the risk of developing several malignancies, in particular NMSC and non‐Hodgkin's lymphoma (Meggitt et al. 2011). Much of the increased risk in this cohort has been attributed to the intensity of immunosuppression (and subsequent susceptibility to oncogenic viruses) rather than to the direct action of azathioprine itself (Karran and Attard 2008). This increased risk of lymphoma (albeit much lower than that after organ transplantation) also appears to occur in other azathioprine‐treated cohorts, in particular IBD with a fourfold increased risk quoted in one meta‐analysis (Kandiel et al. 2005). More recent evidence significantly associates the development of new NMSC in those IBD patients treated with azathioprine for more than 1 year, and increased sensitivity to UV exposure has been implicated in both epidemiological and laboratory‐based studies (Meggitt et al. 2011).

Two studies have demonstrated a slightly increased risk of cancer overall (excluding NMSC) associated with azathioprine therapy in people with MG (Rawoot et al. 2006; Pedersen et al. 2013). The first paper to specifically address the risk of NMSC in azathioprine‐treated MG patients was a Danish case–control study (Pedersen et al. 2014). This study identified a statistically significant increased risk of NMSC in MG patients who had ever used azathioprine and a considerably higher risk in those who had been exposed to a high cumulative dose or long duration of therapy. The results of our study echo these findings, with an incidence rate of NMSC in azathioprine‐treated MG patients just under 16 times that of the incidence in the general Irish population. Despite half of all patients on established azathioprine treatment having been exposed to either a high cumulative dose or long duration of treatment, documented evidence of a discussion about the risk of NMSC development and/or sun exposure‐related preventative measures was only found in three azathioprine‐treated cases. This is in contrast to documented advice about the risk of myelosuppression and/or the need for regular blood test monitoring which was found in almost 80% of cases as well as the practice of TPMT testing prior to treatment commencement in over 40% of cases.

The frequency of adverse effects other than NMSC encountered with azathioprine treatment in this study is in general comparable to rates described in early trial data and large case series of IBD patients (little data exist on azathioprine adverse effect profiles in MG). The percentage of those ever treated with azathioprine developing manifestations of myelotoxicity in this study was 23.8%, which falls within the range of 5–30% (mean 19%) described in a large analysis of 10 studies (Anstey et al. 1992). Drug‐induced liver blood test derangement was encountered in 16.7% cases in our cohort, which is markedly higher than the rate of azathioprine‐induced liver disorders (mean 3.4%) in a systematic review of 3485 patients (Gisbert et al. 2007), however the lack of universally standardized definitions of hepatotoxicity and the absence of established follow‐up intervals could account in part for this discrepancy. A lack of a standardized definition of azathioprine‐induced hypersensitivity and a paucity of data on the development of this adverse effect preclude accurate comparison; however, the rate of hypersensitivity reactions detected in a prospective series of patients treated for ectopic eczema was 6.3% (Meggitt et al. 2011). The combined percentage of patients who developed a true hypersensitivity reaction and those with flu‐like symptoms such as nausea, fever, myalgia, and arthralgia (which can be manifestations of drug‐induced hypersensitivity; Meggitt et al. 2011) in our patient population was 7.2%.

This study has a number of drawbacks, mainly related to use of retrospective data and the potential for confounding bias. No information on sunlight exposure and prior knowledge of the importance of UV exposure and NMSC risk was obtained. Furthermore, patients on high‐dose/long‐duration azathioprine therapy are likely to attend hospital more regularly and may have a higher detection rate of NMSC. It is also of importance to note that high rates of extrathymic neoplasms have been demonstrated in patients with MG regardless of the use of immunosuppressive therapy (Citterio et al. 2009; Levin et al. 2005; Liu et al. 2012). Given the lack of both a clear temporal association between MG and cancer development and a relationship of MG to specific tumors, it has been suggested that immune dysregulation in general rather than a paraneoplastic mechanism may be responsible (Levin et al. 2012). Similarly, an inherent risk of cancer (particularly lymphoma) in conditions such as IBD and rheumatoid arthritis has been identified as a possible confounder in the assessment of the potential association of azathioprine use and neoplasia development (Aithal and Mansfield 2001; Kandiel et al. 2005). Whether this baseline increased risk applies to NMSC in MG has not been established; the incidence rate of 4.6 per 1000 person‐years among MG patients never treated with azathioprine in the Danish case–control study was based on only 14 unexposed cases (Pedersen et al. 2014). In any case, it is impossible to out rule this as a source of confounding bias, particularly as those considered for azathioprine treatment are likely to have more severe disease and hence the state of immune dysregulation may be enhanced.

As outlined in the latest U.S. Preventive Services Task Force recommendation, epidemiological evidence links ultraviolet radiation exposure with incidence of NMSC, preventative measures such as daily sunscreen use have been shown to reduce the incidence of SCC and there is some evidence that counseling on sun‐protective behaviors may be of benefit in the general population (Moyer 2012). In addition, the British Association of Dermatologists' guidelines on azathioprine use stress the importance of discussing the potential increased risk of malignancy with long‐term use as well as giving advice on sun‐protective behaviors (Meggitt et al. 2011). The role of regular dermatological screening in high‐risk patients (i.e., those patients being exposed to a high cumulative dose/long duration of therapy or those with other risk factors for NMSC) is not addressed in equivalent guidelines in nondermatological specialties (Mowat et al. 2011) and has not yet been examined to the best of our knowledge. Given the high incidence of NMSC in the azathioprine‐exposed cohort in this study and recent research in Denmark described above which suggests a considerably increased risk and potential causal association, vigilance among treating neurologists, and a low threshold for dermatology referral is warranted. The results of this study also demonstrate a very low rate of advice provision about NMSC risk in azathioprine‐treated MG patients and thus highlight the need for increased awareness among treating neurologists and patients.

## Conflict of Interest

None declared.

## Note

1 Five patients demonstrated both AChR (acetylcholine receptor) and anti‐striated muscle antibodies. Of those antibody‐negative myasthenia patients, all except one patient were tested for both AChR and MuSK (muscle‐specific receptor tyrosine kinase) antibodies (one was tested for AChR antibodies alone).
